# Transcriptomic analysis and validation reveal the pathogenesis and a novel biomarker of acute exacerbation of chronic obstructive pulmonary disease

**DOI:** 10.1186/s12931-022-01950-w

**Published:** 2022-02-12

**Authors:** Huijie Wang, Yonghong Zhong, Na Li, Min Yu, Lin Zhu, Lina Wang, Fei Chen, Yaping Xu, Jian Liu, Huaqiong Huang

**Affiliations:** 1 Key Laboratory of Respiratory Disease of Zhejiang Province, Department of Respiratory and Critical Care Medicine, Second Affiliated Hospital of Zhejiang University School of Medicine, Hangzhou, 310009 Zhejiang China; 2 Tuberculosis Diagnosis and Treatment Center, Affiliated Hangzhou Chest Hospital, Zhejiang University School of Medicine, Hangzhou, 31000 Zhejiang China; 3 Linping Campus, The Second Affiliated Hospital of Zhejiang University School of Medicine, Hangzhou, 311100 China; 4 Zhejiang University-University of Edinburgh Institute (ZJU-UoE Institute), Zhejiang University School of Medicine, International Campus, Zhejiang University, Haining, 314400 China

**Keywords:** Chronic obstructive pulmonary disease, Acute exacerbation, Transcriptomic, Pathogenesis, Biomarkers

## Abstract

**Background:**

Acute exacerbation of chronic obstructive pulmonary disease (AECOPD) is the main factor that leads to the deterioration of the disease. Currently, the diagnosis of AECOPD mainly relies on clinical manifestations, good predictors or biomarkers are lacking. We aim to reveal specific biomarkers and potential pathogenesis of AECOPD and provide a research basis for the diagnosis and treatment.

**Methods:**

Four patients with AECOPD, four patients with stable COPD, and five control subjects were enrolled for RNA sequencing and KEGG analysis. The mRNA level of target genes was verified by quantitative real-time PCR (qPCR) with an expanded sample size (30 patients with AECOPD, 27 patients with stable COPD, and 35 control subjects). ELISA and immunofluorescence were used to identify the target proteins. Furthermore, the expression and function of WNT/β-catenin signaling pathway were assessed in animal models of COPD.

**Results:**

RNA sequencing showed that 54 genes were up-regulated and 111 genes were down-regulated in the AECOPD. Differentially expressed genes were mainly enriched in WNT signaling pathway, et al. QPCR revealed that multi-genes of the WNT/β-catenin signaling were significantly down-regulated in AECOPD (*P* < 0.05), and β-catenin protein was significantly decreased in plasma of AECOPD and stable COPD (*P* < 0.01), while phosphorylated β-catenin was significantly up-regulated in peripheral blood mononuclear cells of AECOPD (*P* < 0.05). Similarly, WNT ligands, WNT receptors, and downstream signaling molecules were down-regulated, with an increased phosphorylated β-catenin protein in animal models of COPD. Activation of WNT/β-catenin signaling pathway by lithium chloride reduced the expression of phosphorylated β-catenin and ameliorated the COPD-like airway inflammation in mice.

**Conclusion:**

WNT/β-catenin signaling pathway is down-regulated in AECOPD patients and in animal models of COPD. Increased expression of phosphorylated β-catenin in the blood might be a potential biomarker of AECOPD. Activation of WNT/β-catenin pathway may also represent a therapeutic target for AECOPD.

**Supplementary Information:**

The online version contains supplementary material available at 10.1186/s12931-022-01950-w.

## Introduction

Chronic obstructive pulmonary disease (COPD), characterized by persistent respiratory symptoms and restricted airflow, is a chronic airway disease that seriously endangers human health [1]. With continuous exposure to cigarette smoke and environmental pollutants, as well as the aging population, COPD has become the third leading cause of death in the world, with a global prevalence rate of 10.1% [2]. In China, the overall prevalence of COPD in people over 20 years old is 8.6%, in people over 40 years old, a higher prevalence of COPD was 13.7% [3].

Acute exacerbation of chronic obstructive pulmonary disease (AECOPD) is defined as patients who have acute exacerbation of respiratory symptoms and require additional treatment [1]. Frequent acute exacerbation will accelerate the deterioration of lung function and increase the social and economic burden. Predicting and identifying AECOPD is an important part of COPD management. Clinically, the diagnosis of AECOPD mainly depends on medical history and clinical manifestations. In recent years, the biomarkers of COPD have been an appealing direction of research, to find a convenient method for early monitoring, auxiliary diagnosis, and evaluation of treatment response.

Transcriptomics can simultaneously detect thousands of RNA and has gradually become an important tool for biomarker research. RNA sequencing (RNA-seq) is a transcriptomic analysis method using deep sequencing technology, which can detect the overall transcription activity of cells or tissues more accurately, efficiently, and widely [4]. Currently, a variety of RNA and molecular signaling pathways related to COPD were identified by transcriptomic, but the results of different studies are not highly repetitive. It is very important to verify the results of omics in multiple levels for finding reliable signaling pathways and biomarkers.

In this study, we performed RNA-seq of mRNA in peripheral blood mononuclear cells (PBMC) of patients with AECOPD, stable COPD, and control subjects, screened differentially expressed genes (DEGs) and multiple signaling pathways that potentially related to the pathogenesis of AECOPD by KEGG analysis. We use quantitative real-time PCR (qPCR) to verify the transcription level of WNT/β-catenin signaling in an expanded-sample size, use ELISA and immunofluorescence to verify the differential expression of target genes at the protein level. Besides, we further elucidated the expression of WNT/β-catenin signaling pathway in a traditional COPD model with cigarette smoke (CS) exposure, and in a novel COPD model combining cigarette smoke sensitization and elastin challenge (CE).

## Methods

### Patient population

Patients with stable COPD and AECOPD and control subjects were recruited from the Second Affiliated Hospital of Zhejiang University School of Medicine, Hangzhou, China. All participants have given written informed consent to participate in the study, which was approved by the Institutional Review Board for Human Studies of Second Affiliated Hospital of Zhejiang University School of Medicine (Hangzhou, China). The definition of patients with stable COPD and AECOPD was according to the Global Initiative for Chronic Obstructive Lung Disease (GOLD 2018) [5]. Patients with stable COPD were enrolled based on the following criteria: (1) Over 40 years old. (2) In line with the diagnostic criteria in GOLD 2018. (3) With the ability to sign informed consent. (4) Can be followed up according to the study protocol. Exclusion criteria: (1) with other pulmonary diseases (such as lung cancer, sarcoidosis, tuberculosis, pulmonary fibrosis, cystic fibrosis) and severe α1-antitrypsin deficiency. (2) With other inflammatory diseases (such as rheumatoid arthritis and inflammatory bowel disease, etc.). (3) A history of pulmonary surgery, or a diagnosis of malignant tumor recently. (4) A history of blood transfusion in 4 weeks. (5) Participating in a double-blind drug clinical trial. (6) Unable to walk. (7) Using oral or intravenous steroid therapy. (8) With acute exacerbation in 4 weeks. For AECOPD, patients who were over 40 years old were eligible for inclusion if they had a diagnosis of COPD in their primary care clinical record and were presenting with an acute exacerbation of respiratory symptoms. Other specific diseases that may account for sudden changes in respiratory symptoms should be excluded by clinical or laboratory tests. The remaining exclusion criteria were consistent with the above five criteria in stable COPD. The control group included healthy people over 40 years old, excluding those with respiratory symptoms, chronic airway diseases, and other lung diseases, as well as heart, liver, kidney, and other important organ diseases.

### Animal models of COPD

Six to 8 weeks male C57BL/6 mice were purchased from the Animal Centre of Slaccas (Shanghai, China). All mice were maintained in the animal facility of the laboratory animal center of Zhejiang University. Mice at the end of modeling were anesthetized by intraperitoneal injection of 0.2 mL 2% pentobarbital sodium and were killed by cervical dislocation, in the scientific research building of Zhejiang University. All experimental protocols were approved by the Ethical Committee for Animal Studies at Zhejiang University.

CE model: Mice (n = 5) were exposed to CS in a stainless-steel chamber using a whole-body smoke exposure system (TE-10, Teague Enterprises, Woodland, CA, USA) for approximately 2.5 h per day (100 cigarettes), 5 days per week, and last 2 weeks. Control subjects (n = 4) were exposed to filtered room air. Mouse elastin (E6402-SPEC) was purchased from Sigma-Aldrich. 2 mg/ml elastin was suspended in sterile saline and sonicated. 100 μg elastin in 50 μl saline was administered intratracheally for 3 times at day 29, 30, and 31, and mice were sacrificed 48 h after the last elastin challenge. The detailed experimental protocol refers to the previous study [6].

CS model: Mice (n = 6) were exposed to CS in a stainless-steel chamber using a whole-body smoke exposure system (TE-10, Teague Enterprises, Woodland, CA, USA) for approximately 10 times per day (5 cigarettes for each time), 5 days per week, and last 3 months. Control subjects (n = 5) were exposed to filtered room air.

### WNT/β-catenin signaling activation in vivo

The WNT/β-catenin signaling in the lungs from C57BL/6 mice was activated via intraperitoneal injection of lithium chloride (LiCl) (200 mg/Kg/BW/day). LiCl is a substituted compound used as activator to evaluate the participation of WNT/β-catenin signaling pathway. Like canonical WNT ligands, LiCl can suppresses the β-catenin destruction complex (Axin/ APC/ CK1/ GSK3β) by inhibiting GSK-3β [7] (Fig. 3A). LiCl was diluted in sterile water and the fresh stock was prepared for every injection. CE model or air control subjects were treated with LiCl or normal saline (NS) for 5 times at day 29, 30, 31, 32 and 33 (Air + NS, n = 5; CE + NS, n = 4; Air + LiCl, n = 5; CE + LiCl, n = 6). Then mice were sacrificed 5 h after the last LiCl injection.

### RNA sequencing and data analysis

Total RNA from PBMC of four patients with AECOPD, four patients with stable COPD, and five control subjects was extracted using RNAiso Reagent (TaKaRa Company, Dalian, China) according to the manufacturer’s instructions. 2 μg RNA per sample was used as input material for the RNA sample preparations. Sequencing libraries were generated using NEBNext^®^ Ultra™ RNA Library Prep Kit for Illumina^®^ (#E7530L, NEB, USA) following the manufacturer’s recommendations and index codes were added to attribute sequences to each sample. Briefly, mRNA was purified from total RNA using poly-T oligo-attached magnetic beads. Fragmentation was carried out using divalent cations under elevated temperature in NEBNext First Strand Synthesis Reaction Buffer (5X). cDNA was synthesized by reverse transcription with a reverse transcriptase kit (Toyobo, Japan). The library fragments were purified with QiaQuick PCR kits and elution with EB buffer, then end repair, A-tailing, and adapter ligation were implemented. The aimed products were retrieved by agarose gel electrophoresis and PCR was performed, then the library was completed. Transcriptome sequencing was performed on the Illumina Hiseq xTen platform.

Raw Data was processed with Perl scripts to ensure the quality of data used in further analysis. DESeq (v1.16) was used for differential gene expression analysis between two samples with biological replicates using a model based on the negative binomial distribution. The p-value could be assigned to each gene and adjusted by Benjamini and Hochberg’s approach for controlling the false discovery rate. Genes with q ≤ 0.05 and|log_2_Ratio|≥ 1 are identified as DEGs. The KEGG (Kyoto Encyclopedia of Genes and Genomes, http://www.kegg.jp/) enrichment of DEGs was implemented by the hypergeometric test, in which p-value was adjusted by multiple comparisons as q-value, which less than 0.05 was considered as significant enrichment.

### Quantitative real-time PCR

Total RNA from PBMC of 30 patients with AECOPD, 27 patients with stable COPD, and 35 control subjects or the lungs of mice was extracted using RNAiso Reagent (TaKaRa Company, Dalian, China) according to the manufacturer’s instructions. Reverse transcription was performed with a reverse transcriptase kit (Toyobo, Japan). Real-time PCR analysis of 9 human genes (*LGR6*, *FZD4*, and *CTNNB1*, etc.) and 11 mouse genes (*LGR6, FZD4*, and *CTNNB1*, etc.) were measured using specific primers (see Additional file 1) and gene expression assays on the ABI ViiA 7 Real Time PCR System (Applied Biosystems, United States of America). 2^−ΔΔCT^ values was calculated as the relative mRNA level of genes.

### ELISA

The concentration of β-catenin (*CTNNB1*) (BioVision, # K3381-100) in human plasma was quantified using ELISA Kit according to the manufacturer’s protocol.

### Immunofluorescence

Immunofluorescent staining was used to quantify phosphorylated β-catenin (p-β-catenin) in the PBMC of human and lung tissue of mice. PBMC was isolated according to previous study [6], and fixed on the slide with a cytospin (Thermo Fisher Scientific, Cheshire, UK). PBMC and lung tissue were stained using the primary antibodies, p-β-catenin (SATTA CRUZ, sc-57535) and secondary antibodies. And then counterstained with DAPI. Slides were imaged under fluorescent microscopy with the appropriate excitation and emission filter, digitally recorded. The specific fluorescence was analyzed using Case Viewer (2.4.0). The proportion of fluorescent positive cells in each photo of PBMC was counted, and the mean value was used to represent the relative number of positive p-β-catenin cells in each slide. Each scanogram of lung tissue was divided into 4–7 sections with equal area centered on the airway, and the average score was obtained according to the distribution of fluorescence positive cells. Relative positive p-β-catenin cells were analyzed at 10 × images by a blinded participant using a 5-point scale as follows: airway surrounded by a monolayer of positive cells—1, airway surrounded by multiple layers of localized scattered positive cells—2, airway surrounded by multiple layers of extensive scattered positive cells or some of the cells are clustered (sparse)—3, airway surrounded by multiple layers of positive cells with some of the cells are clustered (dense)—4, airway surrounded by multiple layers of extensive positive cells with multiple positive cell masses (dense)—5.

### Western blot analysis

The lung tissue was prepared with RIPA buffer (BL504A, Biosharp, Shanghai, China) containing protease inhibitors (BL507A, Biosharp, Shanghai, China). The supernatants of lung tissue were run on gels and incubated with relevant antibodies, p-β-catenin (SATTA CRUZ, sc-57535). Actin was used as a loading control. Quantification was performed by densitometry and analyzed using Image Studio Lite (LI-COR Biosciences, Lincoln, NE, USA).

### H&E staining

Morphological analysis of lung tissue sections was done by H&E staining (American Mastertech stain kits, #KTHNEPT), following manufacturer recommendations. Histological changes were analyzed under microscopy at 20×.

### Statistical analysis

Data were analyzed using GraphPad Prism 8.0 (GraphPad Software, La Jolla, CA, USA). The Student’s t-test was used to determine the relationship between two groups. One-way analysis of variance (ANOVA) with Newman-keuls test was used to analyze the statistical differences among three or more groups. Data are expressed as mean ± SEM and *P* < 0.05 was considered statistically significant.

## Results

### Demographic characteristics

We used 13 subjects to analyze the DEGs by RNA-seq. Basic demographic characteristics for control subjects, patients with stable COPD or AECOPD are summarized in Table 1. The patient samples were matched for age, gender, smoking history, body mass index (BMI) and basic disease. The forced expiratory volume in 1 s (FEV_1_, % Predicted) in stable COPD and AECOPD were 61.58 ± 11.19 (%) and 45.23 ± 20.97 (%), respectively, with no significant differences. Forced expiratory volume in 1 s/ forced vital capacity (FEV_1_/FVC, %) in stable COPD and AECOPD were 57.46 ± 13.47 (%) and 49.10 ± 15.35 (%), respectively, with no significant differences. The control subjects had no respiratory symptoms and no previous chronic airway disease.

**Table 1:** Demographic of included subjects used for RNA-seq

	Control	Stable COPD	AECOPD
Samples, N	5	4	4
Age, years	76.80 ± 8.66	68.50 ± 5.41	74.00 ± 3.39
Sex	4 male, 1 female	4 male	3 male, 1 female
Smoking status	1 current, 2 former, 2 never	2 current, 1 former, 1 never	1 current, 2 former, 1 never
BMI, kg/m^2^	23.99 ± 2.95	23.54 ± 2.52	22.85 ± 3.90
FEV_1_, %Predicted	NA	61.58 ± 11.19	45.23 ± 20.97
FEV_1_/FVC ratio, %	NA	57.46 ± 13.47	49.10 ± 15.35
Basic disease	Hypertension, diabetes	Hypertension, coronary heart disease	Hypertension, coronary heart disease

*COPD* chronic obstructive pulmonary disease, *AECOPD* acute exacerbation of chronic obstructive pulmonary disease, *BMI* body mass index, *NA* not applicable

Then, 92 subjects were used for qPCR verification, including 35 control subjects, 27 patients with stable COPD and 30 patients with AECOPD (Table 2). The patient samples were matched for age, current smoking, BMI and basic disease. FEV_1_ /Predicted in stable COPD and AECOPD were 53.33 ± 14.64 (%) and 54.71 ± 26.09 (%), respectively, with no significant differences. FEV_1_/FVC in stable COPD and AECOPD was 51.70 ± 11.18 (%) and 49.05 ± 14.58 (%), respectively, with no significant differences. The control subjects had no respiratory symptoms and no previous chronic airway disease.

**Table 2:** Demographics of independent cohort used for validation

	Control	Stable COPD	AECOPD
Samples, N	35	27	30
Age, years	67.66 ± 7.80	68.00 ± 8.00	74.17 ± 7.59
Sex	25 male, 10 female	27 male	29 male, 1 female
Smoking status	13 current, 12 former, 10 never	11 current, 11 former, 5 never	10 current, 18 former, 2 never
BMI, kg/m^2^	24.97 ± 3.08	22.93 ± 2.52	21.42 ± 3.22
FEV_1_, %Predicted	NA	53.33 ± 14.64	54.71 ± 26.09
FEV_1_/FVC ratio, %	NA	51.70 ± 11.18	49.05 ± 14.58
Basic disease	Hypertension, diabetes	Hypertension, coronary heart disease	Hypertension, coronary heart disease

*COPD* chronic obstructive pulmonary disease, *AECOPD* acute exacerbation of chronic obstructive pulmonary disease, *BMI* body mass index, *NA* not applicable

### Multiple AECOPD-related DEGs identified by RNA-seq

**Fig. 1:**
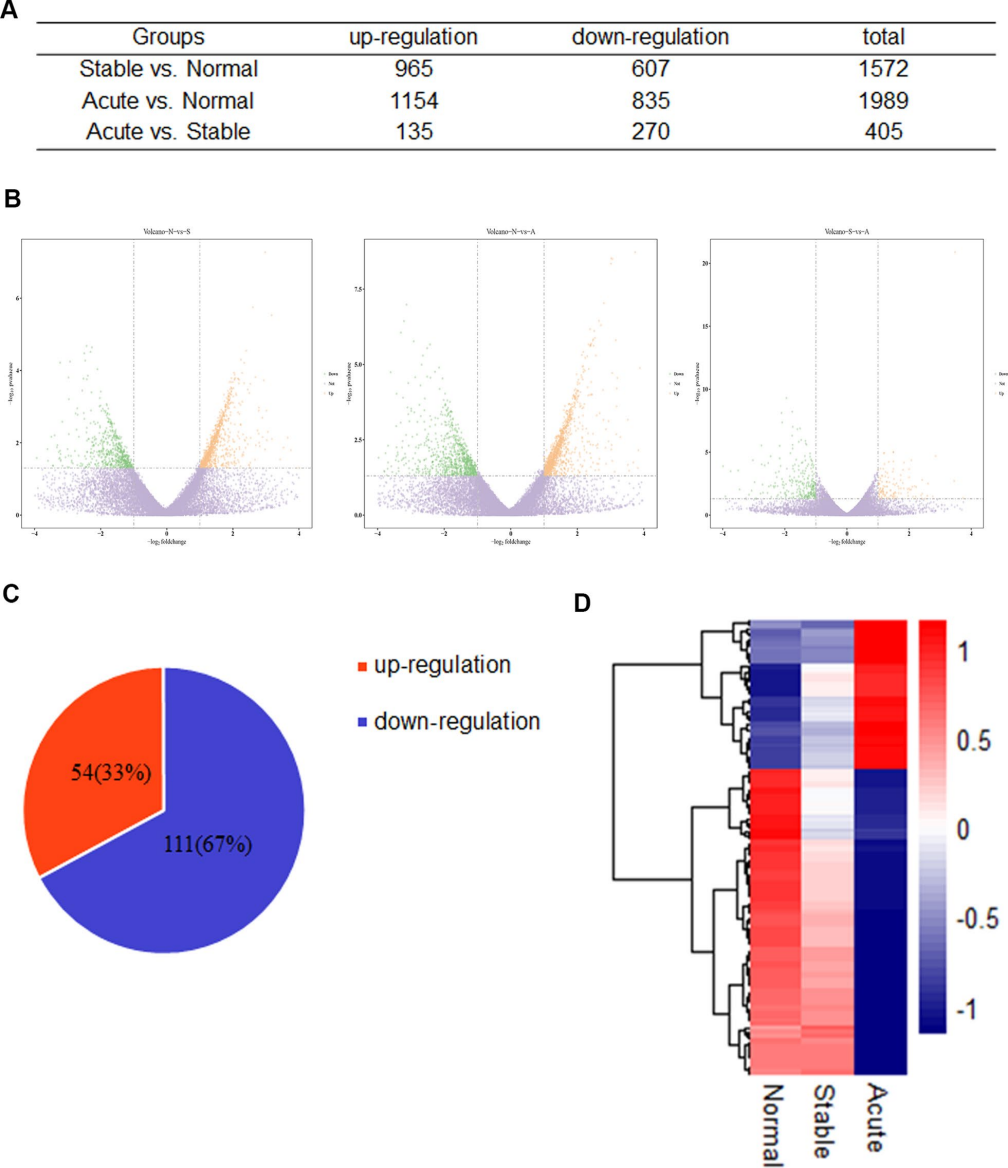
AECOPD-related DEGs identified by differential expression analysis. **A** The number of DEGs between the stable COPD group and the control group, the AECOPD group and the control group, the AECOPD group and the stable COPD group, respectively. **B** The Volcano Plot of DEGs. Each point in the Volcano Plot represents a gene, the horizontal axis represents the − log_2_ (fold change) of a gene expression in two groups. − log_10_ (p value) as the ordinate represents the statistical significance of the change in gene expression. The yellow dots, green dots and purple dots represent up-regulated, down-regulated and non-differentially expressed genes, respectively. **C** According to the disease progression to screen DEGs. **D** The clustering heat maps of DEGs. The abscissa represents the three groups, the ordinate represents the DEGs and the clustering results of the genes. The color represents the gene expression level, the deeper the red color, the higher the gene expression level

The genes with twofold difference in gene expression and *P* value less than 0.05 were regarded as DEGs. We identified 1572 genes significantly differentially expressed between the stable COPD group and the control group, 1989 genes between the AECOPD group and the control group, and 405 genes between the AECOPD group and the stable COPD group. The number of up-regulated and down-regulated DEGs between groups was shown in Fig. 1A, B. According to the characteristics of progression from stable phase to acute exacerbation of COPD, we further screened the up-regulated and down-regulated DEGs in AECOPD. Screening criteria for up-regulated DEGs: The gene expression in the control group was 1.1 times less than that in the stable COPD group, and the gene expression in the AECOPD group was significantly higher than that in the stable COPD group. Screening criteria for down-regulated DEGs: The gene expression in the control group was 0.9 times more than that in the stable COPD group, and the gene expression in the AECOPD group was significantly lower than that in the stable COPD group. According to the screening criteria, a total of 54 up-regulated genes and 111 down-regulated genes in the AECOPD group were obtained (Fig. 1C). With the FPKM value of DEGs as the expression level, a hierarchical clustering analysis was conducted. There were significant differences in gene expression profiles among the three groups, indicating that the changes in gene expression levels were related to the pathogenesis of AECOPD. The regions with different distances represent different gene clustering information, and genes with similar expression patterns may have similar functions or participate in the same biological process (Fig. 1D).

### AECOPD-related signaling pathways obtained by KEGG pathway analysis

**Fig. 2:**
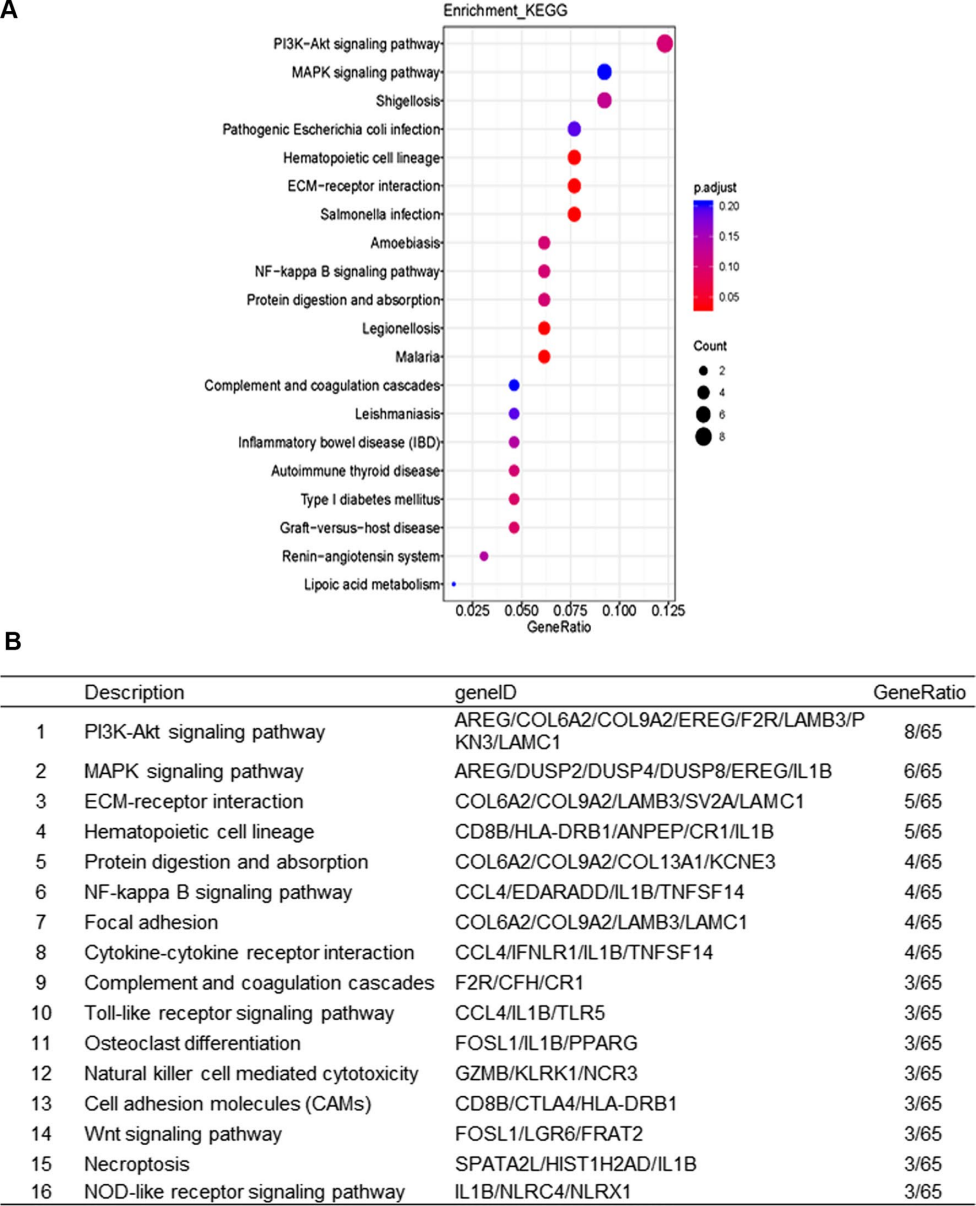
AECOPD-related signaling pathways obtained by KEGG analysis. **A** KEGG pathway enrichment analysis of DEGs. The vertical axis represents signaling pathways. The horizontal axis represents the gene ratio, which is, the number of DEGs to the number of annotated genes in pathways. The size of the dot indicates the number of DEGs in the pathway, and the color of the dot corresponds to different adjusted p value ranges. **B** The pathways and DEGs associated with the pathogenesis of AECOPD. Selection criteria: 1. The number of DEGs enriched in pathways is not less than three. 2. Exclude specific pathogen infection, and disease pathways

To clarify the signaling pathways in which the DEGs were involved, KEGG pathway enrichment analysis was conducted. The top 20 pathways are shown in Fig. 2A. Multiple AECOPD-related signaling pathways were further obtained by using the filter conditions: the number of gene enrichment in the pathway is no less than three, and obvious pathogen infection-related pathways and other disease pathways were excluded. We identified 16 signaling pathways that were potentially associated with the pathogenesis of AECOPD, including phosphoinositide 3-kinase/serine threonine-protein kinase (PI3K/Akt) signaling pathway, mitogen-activated protein kinases (MAPK) signaling pathway, extracellular matrix (ECM)-receptor interaction, and WNT signaling pathway, etc. (Fig. 2B). The RNA-seq results showed that compared with the control group and the stable COPD group, the expressions of *LGR6* and *FOSL1* in the WNT pathway were significantly down-regulated, and the expressions of *FRAT2* were significantly up-regulated in the AECOPD group.

### Validation of down-regulated WNT/β-catenin pathway in AECOPD

**Fig. 3:**
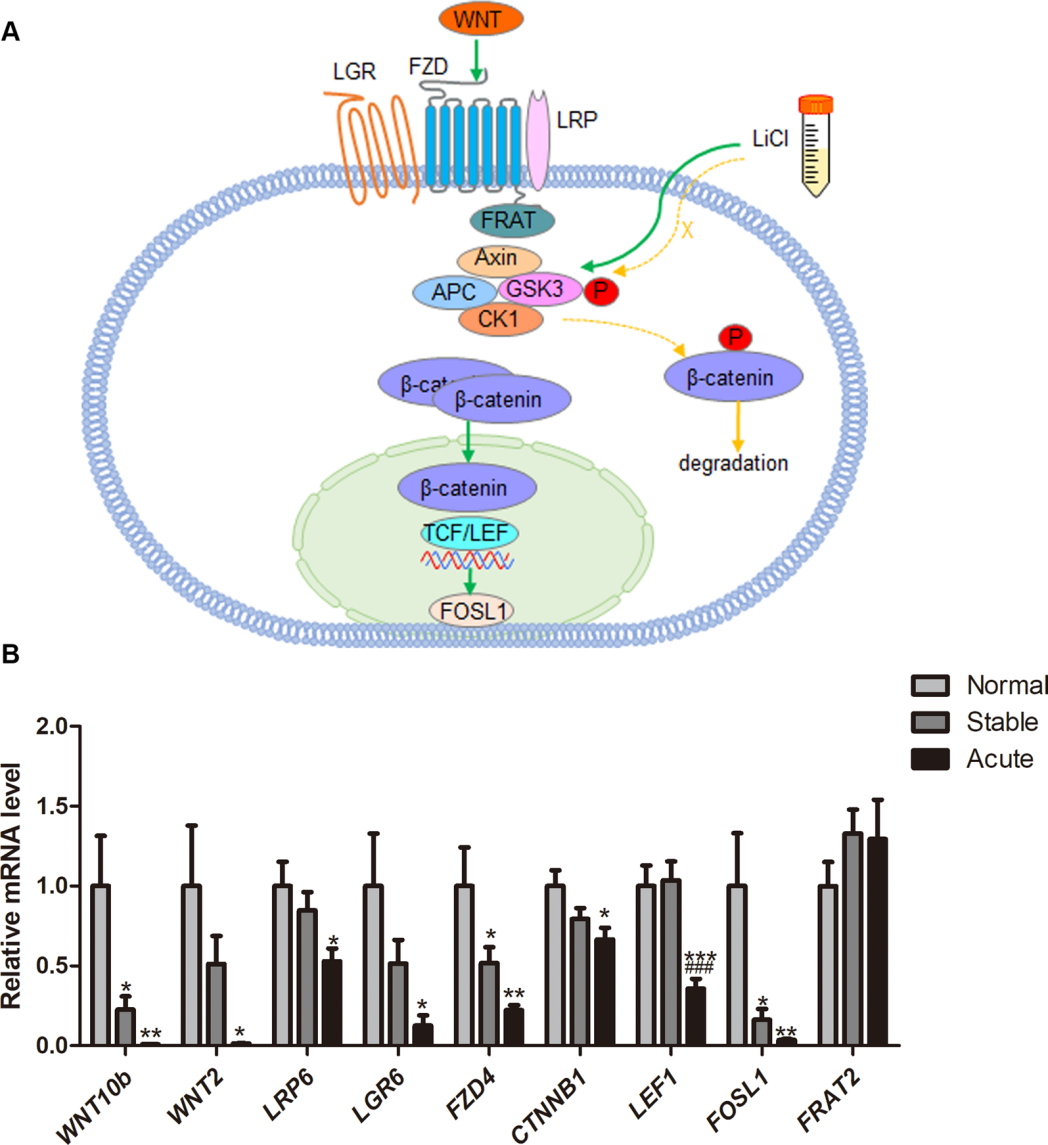
Validation of down-regulated WNT/β-catenin pathway in AECOPD. **A** WNT/β-catenin signaling pathway. The WNT ligands bind to the receptors, including the FZD family, the coreceptor LRP 5/6 and LGR4-6, downstream signaling molecules are activated, the destruction complex (Axin/ APC/ CK1/ GSK-3) is inhibited. The accumulation and translocation of dephosphorylated β-catenin to the nucleus drives the expression of T-cell factor/lymphoid enhancer-binding factor (TCF/LEF)-dependent genes. When there are no WNT ligands or fewer WNT ligands and receptors, the destruction complex is activated, causing phosphorylation of β-catenin to increase and eventually be degraded. Exogenous addition of lithium chloride can also inhibit the destruction complex, promotes β-catenin—mediated gene transcription. **B** The mRNA levels of *WNT10b, WNT2, LRP6, LGR6, FZD4, CTNNB1, LEF1, FOSL1,* and *FRAT2* in the control group (n = 35), the stable COPD group (n = 27) and the AECOPD group (n = 30). Results are presented as relative mRNA level (mean ± SEM). Stable or Acute vs. Normal, *P < 0.05, **P < 0.01, ***P < 0.001 by one-way ANOVA. Acute vs. Stable, ^###^*P* < 0.001 by one-way ANOVA

Previous studies have shown that down-regulation of WNT/β-catenin signaling pathway is associated with impaired lung tissue repair [8–10]. The relationship of WNT/β-catenin signaling genes was shown in Fig. 3A. QPCR of 92 peripheral blood samples (27 patients with stable COPD, 30 patients with AECOPD, and 35 healthy controls) was used to verify the accuracy of RNA-seq results and to elucidate the expression of WNT/β-catenin signaling genes in the blood of AECOPD patients. As depicted in Fig. 3B, compared with the control group, significant down-regulation was observed for *WNT10b, WNT2, LRP6, LGR6, FZD4, CTNNB1, LEF1,* and *FOSL1* in AECOPD (*P* < 0.05), and partial WNT/β-catenin signaling components including *WNT10b, FZD4,* and *FOSL1* were significantly down-regulated in stable COPD (*P* < 0.05). Compared with the stable COPD, *LEF1* was significantly down-regulated in AECOPD (*P* < 0.001). Actually, most of the other genes were further down-regulated in AECOPD, although with no significant difference. Besides, the mRNA level of *FRAT2* among the three groups has no significant difference.

### Expression of WNT/β-catenin pathway proteins in AECOPD

**Fig. 4:**
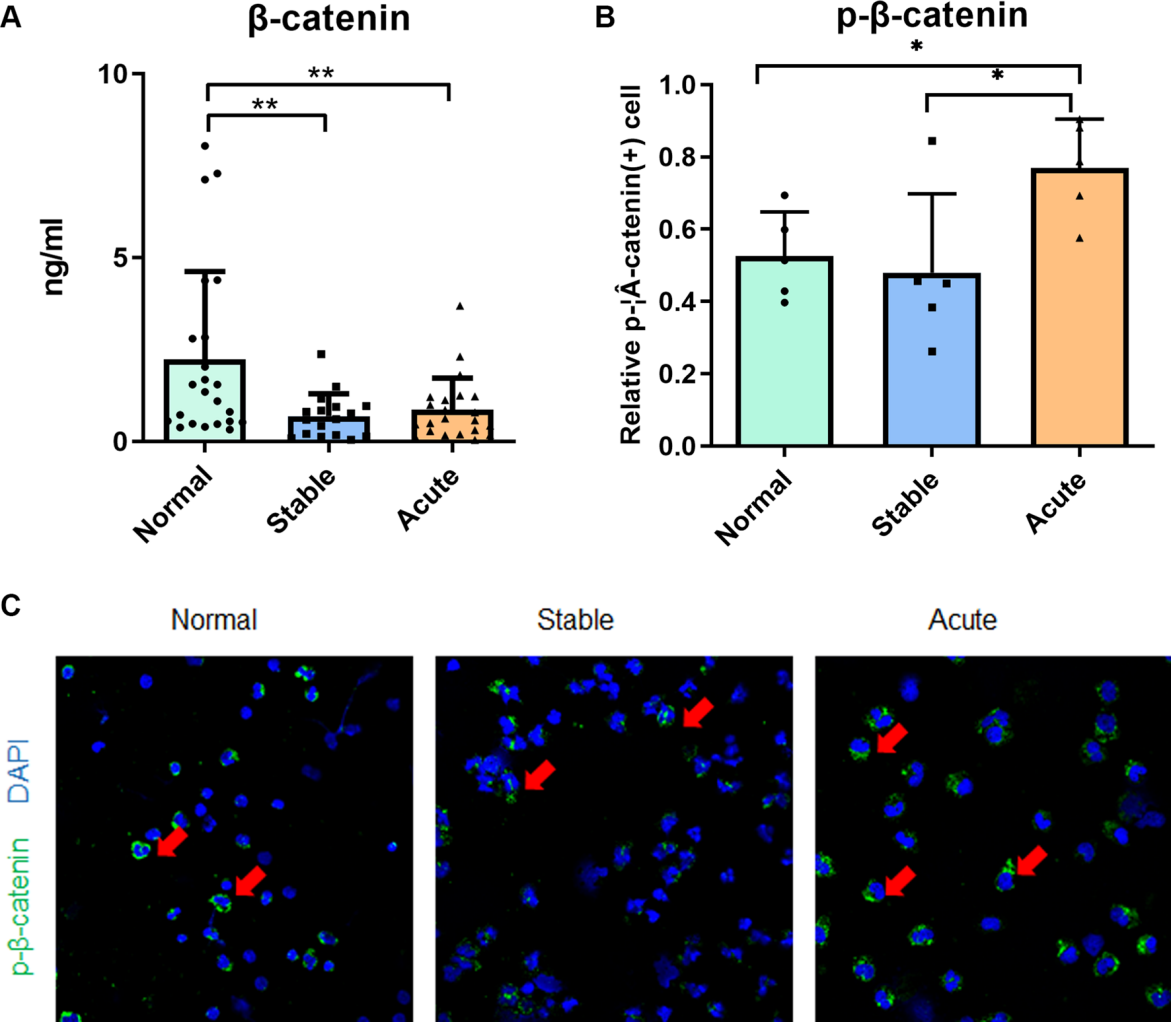
Alterations of β-catenin and its phosphorylation in AECOPD. **A** The expression of β-catenin protein in plasma of control subjects (n = 23), patients with stable COPD (n = 17) and patients with AECOPD (n = 22) was analyzed by ELISA. Results are presented as protein concentration (mean ± SEM). **B, C** The expression of phosphorylated β-catenin protein in PBMC of control subjects (n = 5), patients with stable COPD (n = 5) and patients with AECOPD (n = 5) was analyzed by immunofluorescence. **B** Results are presented as relative number of positive p-β-catenin cells (mean ± SEM). **C** Immunofluorescent staining of PBMC. Green fluorescence represents positive phosphorylated β-catenin cells (red arrowheads). Blue fluorescence represents DAPI. *P < 0.05, **P < 0.01 by one-way ANOVA

To further explore the expression of proteins associated with WNT/β-catenin signaling pathway and search for specific biomarkers of AECOPD, we performed ELISA of β-catenin in plasma and immunofluorescence of p-β-catenin in PBMC. It revealed that β-catenin was significantly down-regulated in stable COPD and AECOPD (*P* < 0.01), but no significant difference between the stable COPD and AECOPD (Fig. 4A). On the other side, p-β-catenin was significantly up-regulated in AECOPD compared with the stable COPD and the control group (*P* < 0.05) (Fig. 4B, C).

### Alterations of WNT/β-catenin pathway in experimental COPD models

**Fig. 5:**
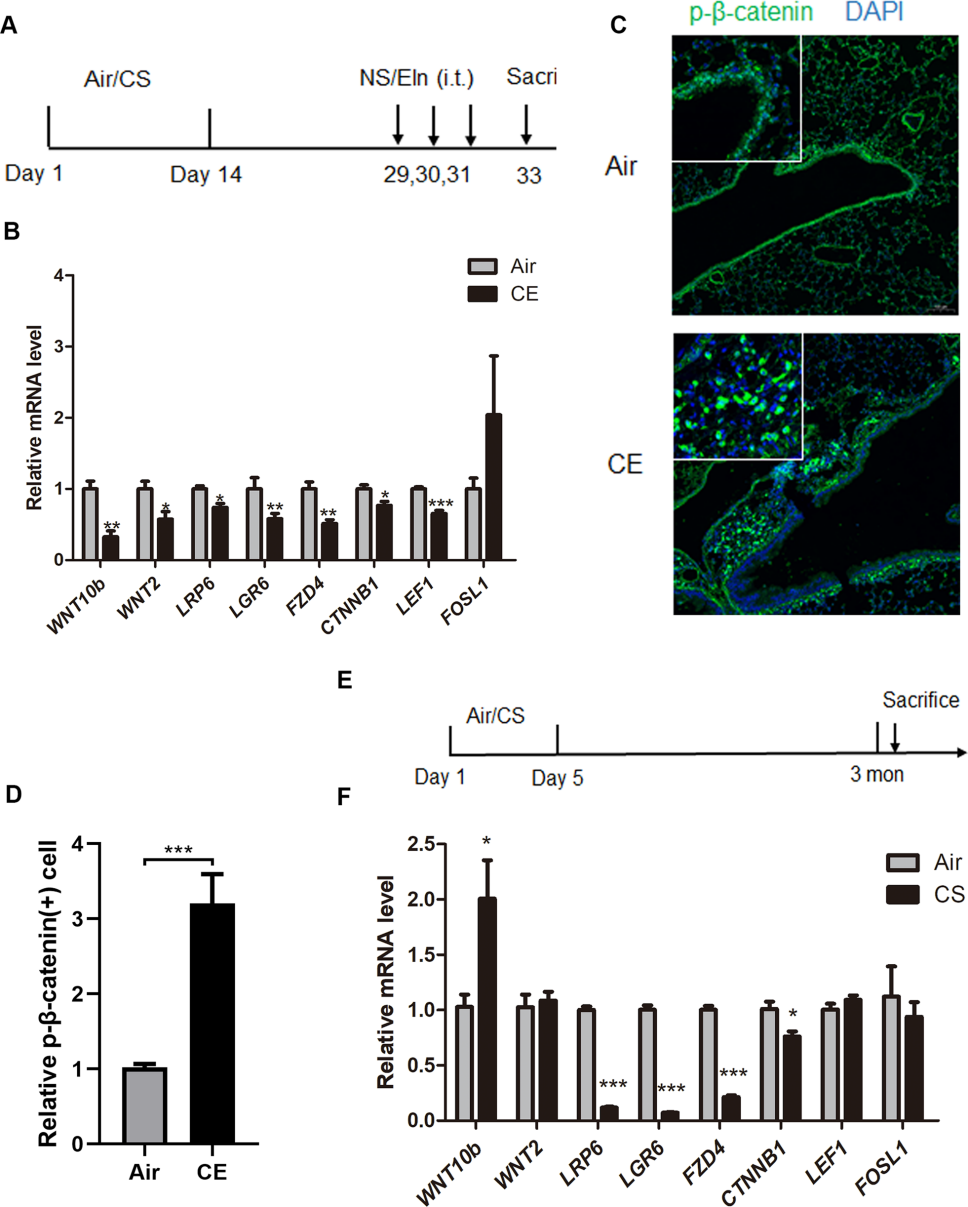
Altered WNT/β-catenin pathway in experimental COPD models. **A–D** CS exposure following elastin challenge in mouse lung tissues. **A** Experimental outline. Mice were exposed to CS (n = 5) or room air (n = 4) for 2 weeks and were hosted at room air for another 2 weeks. Mice were then challenged with elastin (Eln, 100 μg) or normal saline (NS) intratracheally (i.t.) for 3 times at day 29, 30, and 31, and were sacrificed 48 h after the last elastin challenge. **B** The mRNA levels of *WNT10b, WNT2, LRP6, LGR6, FZD4, CTNNB1, LEF1* and *FOSL1* in lung tissue of CE and air control mouse models, were assessed by qPCR. Results are presented as relative mRNA level (mean ± SEM). *P < 0.05, **P < 0.01, ***P < 0.001 by the Student t-test. **C** Representative immunofluorescent staining of lung tissue. Green fluorescence represents positive phosphorylated β-catenin cells. Blue fluorescence represents DAPI. **D** Semi-quantification of positive phosphorylated β-catenin cells. **E, F** CS exposure in mouse lung tissues. **E** Experimental outline. Mice were exposed to cigarette smoke (n = 6) or filtered room air (n = 5), and lung tissue was obtained after 3 months. **F **The mRNA levels of *WNT10b, WNT2, LRP6, LGR6, FZD4, CTNNB1, LEF1,* and *FOSL1* in lung tissue of CS and air control mouse models, were assessed by qPCR. Results are presented as relative mRNA level (mean ± SEM). *P < 0.05, ***P < 0.001 by the Student t-test

The above results indicated that the WNT/β-catenin signaling is down-regulated in the blood of patients with AECOPD. To further elucidate the expression of the WNT/β-catenin signaling pathway in lung tissue, two COPD mouse models were used. Given that there is no reliable mouse model of AECOPD, we referred to an acute inflammation model of cigarette smoke sensitization combining elastin challenge, which showed relatively high levels of neutrophilic inflammation and mucus hyperproduction in airways [6]. Mice were exposed to CS for 2 weeks and were hosted at room air for another 2 weeks, then challenged with elastin intratracheally on three consecutive days (Fig. 5A). Most of the WNT/β-catenin pathway components, including *WNT10b, WNT2, LRP6, LGR6, FZD4, CTNNB1,* and *LEF1,* were significantly down-regulated in CE mice (*P* < 0.05), except *FOSL1* (Fig. 5B). The expression of p-β-catenin protein was significantly up-regulated in CE mice (*P* < 0.05) (Fig. 5C, D).

In the traditional CS-induced COPD model, mice were exposed to CS 5 days a week for 3 months (Fig. 5E). The mRNA of *LRP6, LGR6, FZD4,* and *CTNNB1* was also significantly down-regulated in CS mice (*P* < 0.05). The mRNA of *WNT10b* was significantly up-regulated in CS mice (*P* < 0.05). No significant difference was observed for *WNT2, LEF1,* and *FOSL1* between CS mice and air control mice (Fig. 5F).

### Activation of the WNT/β-catenin pathway reduced phosphorylated-β-catenin and airway inflammation

**Fig. 6:**
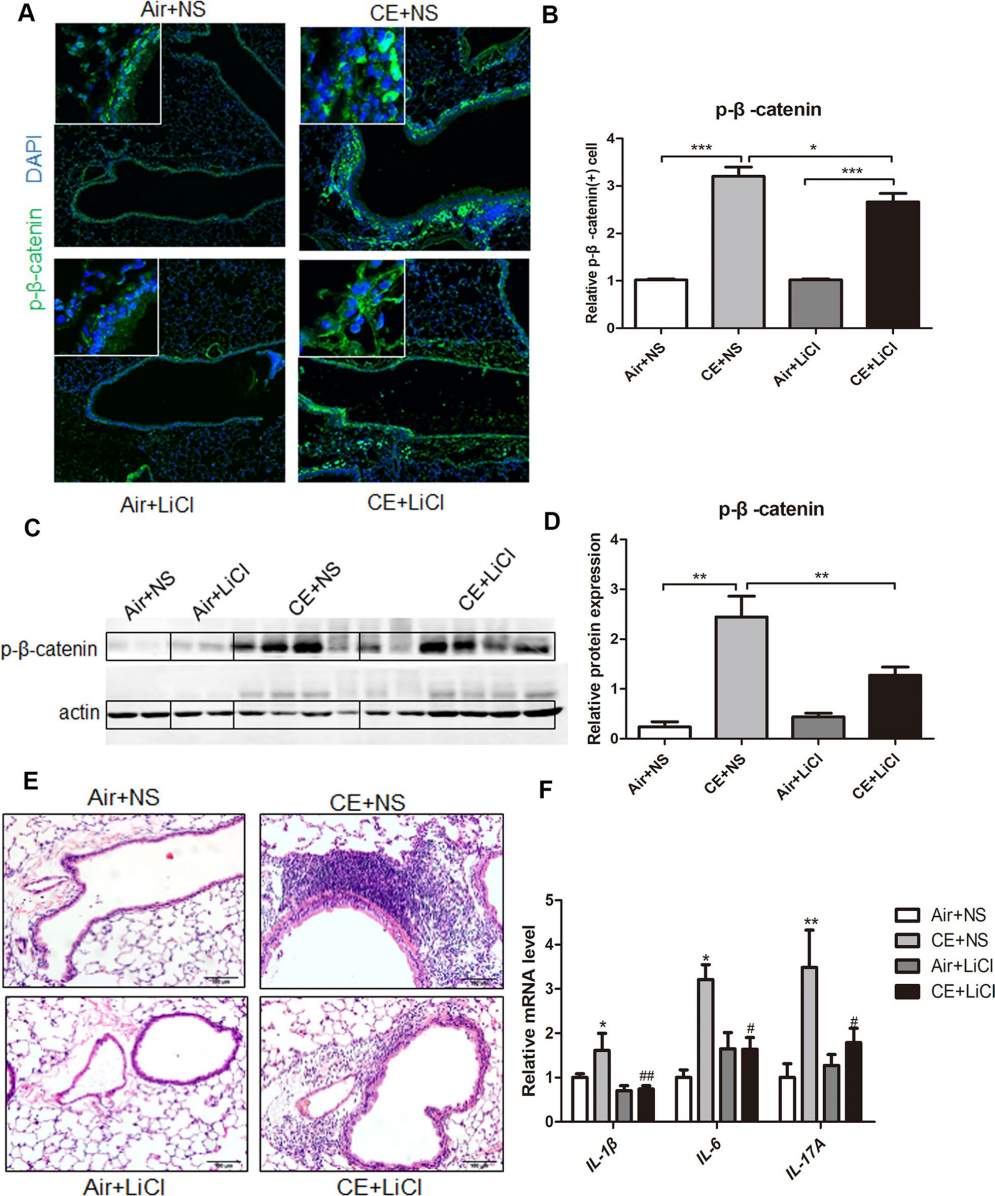
Activation of the WNT pathway reduces phosphorylated-β-catenin and COPD-like airway inflammation. Mice were exposed to CS or room air for 2 weeks and were hosted at room air for another 2 weeks. Mice were then challenged with elastin (Eln, 100 μg) or normal saline (NS) intratracheally (i.t.) for 3 times at day 29, 30, and 31, and were injected with lithium chloride (LiCl) or NS intraperitoneally for 5 times at day 29, 30, 31, 32 and 33 (Air + NS, n = 5; CE + NS, n = 4; Air + LiCl, n = 5; CE + LiCl, n = 6). Then mice were sacrificed 5 h after the last LiCl injection. **A** Representative expression and localization of phosphorylated β-catenin in mice lung tissues by immunofluorescence. **B** Semi-quantitative analysis of the immunofluorescence results. Results are presented as relative number of positive phosphorylated β-catenin cells (mean ± SEM). *P < 0.05, ***P < 0.001 by one-way ANOVA. **C** The expression of phosphorylated β-catenin in mice lung tissue was analyzed by Western Blot. **D** Semi-quantitative analysis of the Western Blot results. Results are presented as relative protein expression concentration (mean ± SEM). **P < 0.01 by one-way ANOVA. **E** H&E staining for histologic assessment of lung tissue. **D** The mRNA levels of *IL-1β, IL-6* and *IL-17A.* Results are presented as relative mRNA level (mean ± SEM). CE + NS vs. Air + NS, *P < 0.05, **P < 0.01; CE + LiCl vs. CE + NS, ^*#*^*P* < 0.05, ^*##*^*P* < 0.01 by one-way ANOVA

Finally, we investigated whether WNT/β-catenin signaling pathway activation could improve the pathological changes of lung tissue. Lithium chloride (LiCl), as a WNT/β-catenin pathway activator, can inhibit the p-β-catenin through GSK3β. The dephosphorylated β-catenin increased in cytoplasm transfers to the nucleus, which mediates downstream signal transduction and gene expression [11]. Immunofluorescence and Western blot analysis of p-β-catenin in the lung tissue revealed that the level of p-β-catenin was significantly up-regulated in CE + NS mice (*P* < 0.01). Importantly, after treatment with LiCl, the level of p-β-catenin was significantly down-regulated in CE + LiCl mice compared with CE + NS mice (*P* < 0.05) (Fig. 6A–D). Moreover, as shown in Fig. 6E, extensive inflammation cells around the airway in CE mice have a remarkable reduction after LiCl injection. Correspondingly, the mRNA level of *IL-1β*, *IL-6*, and *IL-17A* was significantly up-regulated in CE + NS mice compared with Air + NS mice (*P* < 0.05) and was again markedly down-regulated in CE + LiCl mice compared with CE + NS mice (*P* < 0.05) (Fig. 6F).

## Discussion

In this study, RNA-seq was used to obtain DEGs in the blood of different stages of COPD. Combined with bioinformatics analysis, multiple signaling pathways were found to participate in the pathogenesis of AECOPD, which is consistent with previous studies [9, 12–14]. Importantly, we demonstrated, for the first time, that the WNT/β-catenin signaling pathway is decreased in patients with AECOPD. A key component of this pathway p-β-catenin protein is up-regulated in the blood of patients with AECOPD. Down-regulation of WNT/β-catenin signaling pathway was also observed in the lung tissues of experimental COPD models. Activation of WNT/β-catenin signaling pathway can reduce the expression of p-β-catenin protein and alleviate lung inflammation in CE model.

In the present study, RNA-seq was used to identify DEGs in the blood of patients with stable COPD and AECOPD, as well as control subjects. The number of DEGs obtained by RNA-seq was correlated with the number of biological replicates, the sequencing depth and the fold change. Studies have found that when the number of biological replicates was 3, 5 and 10, the statistical power to detect DEGs of twofold change was 87%, 98% and 100%, respectively [15]. For a certain number of biological replicates, increasing the sequencing depth will increase the number of DEGs detected, however, when the sequencing depth was above 10 M, the number of DEGs increased slowly [16]. In this study, we recruited 5 control subjects, 4 patients with stable COPD, and 4 patients with AECOPD for RNA-seq, with a sequencing depth of 40 M and a fold change of 2 times, which can make the statistical power to detect DEGs close to 98%. Moreover, the clinical characteristics of the subjects we recruited were similar. After RNA-seq and data processing, we obtained DEGs with good sequencing quality and high expression levels.

One of the aims of the present study was to identify and annotate the DEGs. Thousands of DEGs were revealed from the PBMC between different groups. And 54 up-regulated genes and 111 down-regulated genes in AECOPD were considered as disease-related genes, according to the characteristics of disease progression from stable to acute exacerbation of COPD. To elucidate the biological processes involved in these genes, we used KEGG database for enrichment analysis and identified 16 signaling pathways that may be related to the pathogenesis of AECOPD. Some previous studies have demonstrated the correlation between the signaling pathways and the pathological process of COPD. PI3K signaling pathway is significantly activated and is associated with increased susceptibility to pulmonary infection in patients with COPD. Cigarette smoke extract can induce Akt degradation, activate the death-signaling pathway, and lead to cell death in normal human lung fibroblasts [12, 17]. ECM protein promotes proliferation, migration, and adhesion of airway smooth muscle cells in COPD rat model by activating PI3K-Akt signaling pathway [18]. Activation of the p38 MAPK pathway appears to be involved in the pathogenesis of COPD, and drug inhibition of P38 MAPK can reduce the secretion of pro-inflammatory factors in alveolar macrophages. In clinical trials, treatment with p38 MAPK inhibitor for 6 weeks can improve FEV_1_ in COPD patients [13, 19, 20].

Except for the above signaling pathways, we found that 3 DEGs enriched in WNT signaling pathway. The WNT family includes 19 glycoproteins, which interact with transmembrane receptors to mediate the signal transduction from cytoplasm to nucleus, thus regulating gene expression, causing cytoskeleton rearrangement, and changing cell polarity [21]. The canonical WNT signaling pathway relies on a key transcriptional coactivator, β-catenin. When the WNT ligands bind to their receptors, including the FZD family, the coreceptor LRP 5/6 and LGR4-6 (as a stabilizer for the two receptors), the destruction complex (Axin/ APC/ CK1/ GSK-3) is inhibited, and the phosphorylation of β-catenin is blocked by GSK-3. The accumulation and translocation of dephosphorylated β-catenin to the nucleus drives the expression of T-cell factor/lymphoid enhancer-binding factor (TCF/LEF)-dependent genes [22, 23]. WNT signaling pathway plays an important role in the development and function of many organ systems, including the respiratory system. Dysfunction of WNT signaling pathway is related to the occurrence and development of many lung diseases, such as asthma, COPD, idiopathic pulmonary fibrosis, and non-small cell lung cancer. In studies on WNT signaling pathway and COPD, it has been demonstrated that WNT/β-catenin pathway is involved in lung epithelial injury and repair, and the reduction of WNT/β-catenin signaling pathway is associated with emphysema parenchymal tissue destruction and impaired repair capacity [9]. The WNT receptor FZD4 is decreased in human and experimental COPD which contributes to impaired alveolar repair capacity [8]. Considering the important role of WNT/β-catenin pathway in stable COPD, whether it is involved in the pathological process of AECOPD aroused our interest.

Since RNA-Seq was conducted on a relatively small set of patients to reveal the transcriptome profiles, which provided the general understanding of the AECOPD patients compared with the controls and came out the key targets for the following validations and functional investigations. To validate the critical targets identified from our RNA-Seq analysis, validation examined by a more significant number of patients was performed. In addition, it will aid in concluding relatively reliable conclusions on our findings of these main targets. By qPCR, we found that the mRNA expression of WNT ligands (*WNT10b, WNT2*), WNT receptors (*LRP6, LGR6, FZD4*) and downstream signaling molecules (*CTNNB1, LEF1, FOSL1*) were down regulated in the PBMC of AECOPD patients. Importantly, to our knowledge, it is for the first time to identify that the expression of p-β-catenin protein was up-regulated in the PBMC of AECOPD patients, which suggests its potential value as a novel biomarker of AECOPD.

To further evaluate the impact of WNT/β-catenin signaling pathway during acute airway inflammation and stable COPD, we applied two different animal models, CE and CS exposure, respectively. The CE model has been demonstrated with much higher levels of neutrophilic airway inflammation, mucus hyperproduction, and predominant Th17 response than traditional CS model, somehow mimicking the bronchitis phenotype or AECOPD [6, 24, 25]. The down-regulation of WNT/β-catenin signaling pathway was observed in both animal models, which emphasized that the decreased activity of WNT/β-catenin pathway was not only involved in the progression of COPD but might also be associated with the onset of acute exacerbation. Interestingly, activation of WNT/β-catenin pathway by LiCl can reduce the expression of p-β-catenin protein around the airway and improve lung inflammation in CE mice, further suggesting p-β-catenin as a biomarker and a therapeutic target. In recent years, numbers of studies have reported that β-catenin participates in several inflammatory diseases, including acute lung injury [26], chronic rhinosinusitis [27], sepsis [28], osteoarthritis [29] and so on. Moreover, our results are in accordance with a previous publication, reporting that the lung inflammatory induced by cigarette smoke extract and airspace enlargement induced by elastin in mice can be alleviated by the WNT pathway activator, LiCl, and it was associated with increased activation of erythroid-2 related factor-2 pathway [30].

Some limitations of this study are also worth discussing. Firstly, although the sample size of RNA-seq in this study was able to make the detection rate of DEGs close to 98%, further studies with a larger sample size were needed to confirm our results. Secondly, it is worth further investigating the expression of WNT/β-catenin pathway-related proteins in sputum of AECOPD patients. Thirdly, whether the value of p-β-catenin as a potential biomarker of AECOPD is better than clinical indicators still needs to be explored, and it should be noted, immunostaining of p-β-catenin is not as convenient as other common methods like ELISA for its clinical application. Finally, except the WNT/β-catenin pathway, the role of other signaling pathways obtained by RNA-seq in the pathogenesis of AECOPD is worth further exploring.

In conclusion, we find 16 potential signaling pathways related to the pathogenesis of AECOPD, including PI3K/Akt signaling pathway, MAPK signaling pathway, and ECM- receptor interaction, et al. Remarkable, we demonstrated, for the first time, that the WNT/β-catenin signaling pathway is decreased in patients with AECOPD and CE mouse model. Phosphorylated β-catenin protein is up-regulated in the blood of patients with AECOPD. Therapeutic activation of WNT/β-catenin signaling pathway can alleviate lung inflammation in CE mouse model. As a new potential biomarker of AECOPD, p-β-catenin is expected to provide a new research target for the early diagnosis and treatment of AECOPD.

## Supplementary Information

**Additional file 1: Table S1.** The primers sequence for qPCR of human blood. **Table S2.** The primers sequence for qPCR of mice lung tissue.

## Data Availability

The authors confirm that the data supporting the findings of the present study are available within the article.

## Abbreviations

RNA-seq: RNA sequencing
PBMC: Peripheral blood mononuclear cells
DEGs: Differentially expressed genes
CS: Cigarette smoke
CE: Cigarette smoke sensitization and elastin challenge
p-β-catenin: Phosphorylated β-catenin
