# Supplementary material for: Transcriptomic analysis and validation reveal the pathogenesis and a novel biomarker of acute exacerbation of chronic obstructive pulmonary disease
Source: Respir Res. 2022 Feb 12;23:27. doi: 10.1186/s12931-022-01950-w (PMC8840779; doi:10.1186/s12931-022-01950-w)
Supplement: Supplementary file 1 — Additional file 1: Table S1. The primers sequence for qPCR of human blood. Table S2. The primers sequence for qPCR of mice lung tissue. [file 12931_2022_1950_MOESM1_ESM.docx]

**Table S1** **The primers sequence for qPCR of human blood**

| Gene | Primers sequence (5' to 3') | |
| --- | --- | --- |
| *WNT10b* | Forward | GTCCCGAGGCAAGAGTTTCC |
|  | Reverse | GGTTACAGCCACCCCATTCC |
| *WNT2* | Forward | GCCGTGTGTGCAACCTGACT |
|  | Reverse | CACCCACACTTGGTCATCCG |
| *LRP6* | Forward | CCTCGAGCCGTTGTGGTAAA |
|  | Reverse | ACCTCCCGTTCTGTCCCATC |
| *LGR6* | Forward | GGCAGCCATTGTGTAGAGCC |
|  | Reverse | CTCCACCTGCTGGCGTAGAT |
| *FZD4* | Forward | GTGCCAGAACCTCGGCTACA |
|  | Reverse | CCGTACTGGATGAGCGGTGT |
| *CTNNB1* | Forward | CTGCCCTGGTGAAAATGCTT |
|  | Reverse | CGCACTGCCATTTTAGCTCC |
| *LEF1* | Forward | CCCACACAACTGGCATCCCT |
|  | Reverse | ATGCTGAGGCTTCACGTGCA |
| *FOSL1* | Forward | CAGGAGCTGCAGTGGATGGT |
|  | Reverse | CTTCGACGTACCCCTGGAGG |
| *FRAT2* | Forward | ACGACGACCCGCATCGGCTC |
|  | Reverse | GTCAGGTCCGCTGCGGCCTC |
| *GAPDH* | Forward | AGCCACATCGCTCAGACAC |
|  | Reverse | GCCCAATACGACCAAATCC |

**Table S2** **The primers sequence for qPCR of mice lung tissue**

| Gene | Primers sequence (5' to 3') | |
| --- | --- | --- |
| *WNT10b* | Forward | GAAGGGTAGTGGTGAGCAAGA |
|  | Reverse | GGTTACAGCCACCCCATTCC |
| *WNT2* | Forward | CCTCCGAAGTAGTCGGGAATC |
|  | Reverse | GCAGGACTTTAATTCTCCTTGGC |
| *LRP6* | Forward | TTGTTGCTTTATGCAAACAGACG |
|  | Reverse | GTTCGTTTAATGGCTTCTTCGC |
| *LGR6* | Forward | CTGCGCCTAGATGCTAATCTC |
|  | Reverse | GGTGAGTGCATTGTCATCCAG |
| *FZD4* | Forward | AACCTCGGCTACAACGTGAC |
|  | Reverse | GGCACATAAACCGAACAAAGGAA |
| *CTNNB1* | Forward | CCCAGTCCTTCACGCAAGAG |
|  | Reverse | CATCTAGCGTCTCAGGGAACA |
| *LEF1* | Forward | AACGAGTCCGAAATCATCCCA |
|  | Reverse | GCCAGAGTAACTGGAGTAGGA |
| *FOSL1* | Forward | ATGTACCGAGACTACGGGGAA |
|  | Reverse | CTGCTGCTGTCGATGCTTG |
| *IL-1β* | Forward | GAAATGCCACCTTTTGACAGTG |
|  | Reverse | TGGATGCTCTCATCAGGACAG |
| *IL-6* | Forward | CTGCAAGAGACTTCCATCCAG |
|  | Reverse | AGTGGTATAGACAGGTCTGTTGG |
| *IL-17A* | Forward | TCAGCGTGTCCAAACACTGAG |
|  | Reverse | CGCCAAGGGAGTTAAAGACTT |
